# The Role of BSL-3 Laboratories in Pandemic Preparedness: A Focus on Brazil's Infrastructure for Biosafety and Disease Control

**DOI:** 10.1155/tswj/9104904

**Published:** 2025-09-18

**Authors:** Roni Vinhas, Fabricia Oliveira, Larissa Fonseca, Katharine Hodel, Claudio Mafra, Cíntia Minafra, Marilda Gonçalves, Bruna Machado

**Affiliations:** ^1^Gonçalo Moniz Institute (IGM) Oswaldo Cruz Foundation (Fiocruz), Salvador, Brazil; ^2^SENAI CIMATEC University Center, Salvador, Brazil; ^3^SENAI Institute for Advanced Health Systems SENAI/CIMATEC University Center, Salvador, Brazil; ^4^Center for Biological and Health Sciences, Department of Biochemistry and Molecular Biology, Federal University of Viçosa, Viçosa, Minas Gerais, Brazil; ^5^School of Veterinary and Animal Science, Federal University of Goiás, Goiânia, Brazil

**Keywords:** biosafety, BSL-3 laboratories, COVID-19, health surveillance, pandemic, public health

## Abstract

BSL-3 laboratories are fundamental for the safe handling of infectious microorganisms that require high-containment measures. Through a literature review, this work was aimed at highlighting the importance of these laboratories in supporting research and public health responses, especially during health emergencies. The review presents an overview of the global distribution of BSL-3 facilities, the impacts of the COVID-19 pandemic on laboratory investments, and future perspectives on their role in national development. It was observed that the pandemic exposed limitations in laboratory capacity, leading many countries to operate in suboptimal environments, underscoring the need for strict biosafety standards and preparedness infrastructure. This review also identifies disparities in global BSL-3 capacity—particularly in low- and middle-income countries—and examines the Brazilian context, where the absence of a unified regulatory framework hinders progress. By synthesizing international trends and Brazil's recent initiatives, including the development of its first BSL-4 laboratory, this work contributes to understanding the challenges and opportunities for strengthening biosafety infrastructure in support of equitable pandemic preparedness.

## 1. Introduction

Human infectious diseases, even after the advent of vaccines, are still responsible for substantial morbidity and mortality rates. This century has witnessed a wave of severe outbreaks of infectious diseases, which developed into a pandemic with a significant impact on lives and livelihoods, like the COVID-19 pandemic [1]. Cases such as this one call into question the thought that infectious diseases would no longer be a significant public health problem; however, the emergence of new human pathogens and resurgence of infections indicate the continuity of health problems associated with these diseases, as well as point to the need for better structuring and preparation of countries facing outbreaks [2]. Investing in robust infrastructure, such as biosafety level (BSL) laboratories, is paramount for effective disease management and research.

BSL laboratories are classified based on the risk level of the microorganisms handled. As each level progresses, additional considerations are included at the previous level in the form of necessary equipment and infrastructure, aligning the physical design with infectivity, disease severity, transmissibility, and the nature of the work performed [3]. Biosafety Level 1 (BSL-1) works with agents that do not cause human disease. These pose no potential risk to people or the environment; for example, experiments using nonpathogenic *Escherichia coli*. Biosafety Level 2 (BSL-2) works with microorganisms that generate moderate risk for the laboratory and the community; for example, Hepatitis A. Biosafety Level 3 (BSL-3) works with agents that can lead to severe and potentially lethal diseases through the respiratory tract, such as *Mycobacterium tuberculosis* that causes tuberculosis. Finally, Biosafety Level 4 (BSL-4) is the highest BSL, designed for work with agents that may be transmitted via aerosol, which leads to fatal diseases in humans and for which there are no vaccines or treatments available, such as the Ebola virus [4].

Recently, the importance of these facilities gained great notoriety with the onset of the COVID-19 pandemic, due to the demand for a rapid response to the healthcare emergency through suitable locations for the development of research endeavors by those studying the disease, rapid diagnostic and spanning from its identification to the exploration of prevention methods [5]. Private or public healthcare laboratories and research laboratories must constantly demonstrate vigilance regarding the dangers associated with the cultivation and spread of infectious agents [6]. With this in perspective, there is an increasing need for adopting a harmonized approach to biosafety management in places exposed to potential pathogens, as well as in areas and countries that still lack dedicated facilities to address emerging risks. This can directly contribute to addressing global public health concerns [7].

The COVID-19 pandemic highlighted the critical role of BSL-3 facilities in rapid diagnostics, research, and vaccine development [8]. The implementation of these structures, especially in countries that lack this investment, would be of fundamental importance to deal with the emergency risk of this and other respiratory diseases, which are currently drawing attention to the prospects of causing outbreaks and possible outbreaks due to the potential dissemination associated with infection by these types of pathogens [9]. Brazil, for instance, grapples with numerous viral pathogens, including arboviruses, and would significantly benefit from expanded BSL-3 capacity [10].

Given the events of the past, it is possible to show where the health systems have acted successfully. However, it is also possible to identify where there have been failures in facing the outbreaks of respiratory viruses. One of them was the lack of infrastructure in some countries, particularly BSL-3 laboratories, mobile diagnostic units, and genomic surveillance platforms, which led to the adaptation of less suitable environments [11, 12]. This review is aimed at emphasizing the importance of preparedness through strategic investments in BSL-3 laboratories and other essential measures. By doing so, decision-makers and stakeholders can better navigate future health challenges and protect populations from the devastating impact of infectious diseases.

## 2. BSL-3 Laboratories: Structure, Use, and Regulatory Issues

BSL-3 laboratories are specialized facilities meticulously designed for the safe handling of Risk Group 3 microorganisms. These pathogens pose a high individual risk, though the community risk is generally moderate and often has available treatments and preventive measures. BSL-3 laboratories are crucial for research involving infectious agents transmissible through aerosols, which can cause severe or life-threatening diseases in humans or animals [13].

The architecture and engineering controls of a BSL-3 laboratory prioritize the containment of pathogens and the safety of personnel and the surrounding environment. Guidelines and manuals, such as those published by the World Health Organization (WHO) *Laboratory Biosafety Manual* [13], the *CDC Microbiology and Biomedical Laboratory Biosafety Manual* [4], the National Institutes of Health (NIH) *Design Requirements Manual* [14], and the World Organization for Animal Health (OIE) *Terrestrial Animal Health Code* [15], outline stringent requirements for laboratory design and operation. These requirements are aimed at reducing and preventing operator exposure to aerosols, preventing accidental escape of stored or manipulated pathogens, reducing or eliminating the risk of accidents that may lead the operator to direct exposure to the manipulated pathogens, and preventing biosafety violations of pathogens with a severe risk of causing diseases with considerable morbidity and mortality [16].

It is noteworthy that the WHO, in its most recent laboratory biosafety manual, has transitioned away from the traditional BSL terminology [13]. Instead, the WHO now employs a risk group classification system that considers not only the pathogenicity of the microorganism but also factors such as the availability of preventive measures and treatments and the potential for community transmission. This shift reflects a more comprehensive approach to biosafety, acknowledging the multifaceted nature of risk associated with infectious agents.

While high-containment laboratories are vital for pandemic preparedness and research, their proliferation also increases the potential for adverse events, including accidental exposures and intentional misuse of pathogens [17–19]. Implementing robust reliability measures is essential to mitigate these risks. These measures encompass access and inventory controls; surveillance systems; strict protocols for handling, transport, and disposal of microorganisms; and geographic risk assessments.

It is possible to discuss some reliability measures that can be applied to the context of high-containment laboratories, such as establishing access and inventory controls, restricting access to areas involving personal identification systems, and keeping up-to-date records of all biological agents stored, respectively. Monitoring through the implementation of surveillance systems should also be carried out, in addition to other safety procedures to establish clear and strict protocols for the handling, transportation, and disposal of microorganisms. Another measure that should be considered is carrying out an analysis of the geographical distribution of these laboratories, exploring factors such as population density and proximity to urban areas, since this knowledge can help minimize the risk of accidental exposure in the event of failures in the containment system [18, 20].

Regarding the use of protective equipment, for BSL-3, it is essential to have primary barriers (personal protective equipment—PPE) and secondary barriers (architecture, building, installation) to carry out activities safely in the laboratory. PPE such as gloves, lab coats, coveralls, respirators, hoods, face shields, goggles, shoes, or boots constitute the primary barrier, as well as equipment such as boilers for effluent treatment, autoclaves, emergency showers, and eye washers. However, the primary barrier in a BSL-3 environment is the biosafety cabinets (BSCs), equipment used for handling infectious microorganisms, which, if used correctly, will not compromise the protection offered to the user. The secondary barrier is related to the physical infrastructure aiming to protect the environment from infectious materials and provide safe conditions for users in the execution of activities and operations. It is essential to highlight that the air conditioning and exhaust system must be designed for operation with exclusive dedication to functionalities and permanent monitoring of the parameters to provide an adjusted balance of the system based on the airflow (always in a single direction, that is, from the least contaminated area to the most contaminated area) [21].  Table 1 shows a set of the critical requirements to be considered for a BSL-3 facility [21].

BSL-3 laboratories are versatile, serving clinical, diagnostic, teaching, research, and production purposes [22]. They play an indispensable role in diagnosing infectious diseases, understanding pathogen biology, developing vaccines and treatments, producing biological agents, and responding to epidemics and pandemics [10].

It is crucial to acknowledge that despite the existence of WHO guidelines, there is currently no overarching international authority responsible for monitoring or regulating the operation of high-containment facilities such as BSL-3 laboratories. Consequently, the onus of implementing appropriate biosafety policies falls upon individual countries and institutions [23].

In the United States, for instance, two primary regulatory frameworks exist: the Select Agent Program and the NIH Guidelines for Research Involving Recombinant or Synthetic Nucleic Acid Molecules. However, neither program comprehensively covers all BSL-3 activities. The Select Agent Program, administered by the US Department of Agriculture, focuses on agents posing a severe threat to public, animal, or plant health, potentially excluding BSL-3 pathogens deemed to have a lower risk of misuse [23, 24]. The NIH Guidelines for Research Involving Recombinant or Synthetic Nucleic Acid Molecules provide comprehensive requirements for experiments involving such materials. However, these guidelines are primarily focused on recombinant and synthetic nucleic acids and may not explicitly cover all types of biological research conducted in BSL-3 laboratories, particularly those that do not involve genetic manipulation [25]. Likewise, the Federal Select Agent Program regulates a defined list of biological agents and toxins considered to pose a severe threat to public, animal, or plant health [23]. Therefore, BSL-3 activities not involving select agents or recombinant DNA may be subject to institutional biosafety oversight rather than direct federal regulation.

BSL-3 laboratories serve a critical and multifaceted role in combating infectious diseases, from diagnosis to vaccine development. However, the lack of a centralized international regulatory body highlights the current reliance on individual nations and institutions to uphold biosafety standards. The regulatory frameworks in the United States, such as the Select Agent Program and NIH Guidelines, primarily focus on specific categories of high-risk agents and recombinant research [25]. As a result, oversight of BSL-3 laboratories may vary depending on the nature of the work and the institutional biosafety policies in place. As the global landscape of infectious diseases evolves, understanding the distribution and operational status of BSL-3 laboratories worldwide becomes increasingly vital, paving the way for a more comprehensive assessment of global preparedness and potential vulnerabilities.

### 2.1. Identifying BSL-3 Laboratories Worldwide

To estimate the global distribution of BSL-3 laboratories, a 2022 survey conducted by the Center for Security and Emerging Technology (CSET) investigated publications in the PubMed Central database. The study identified 57 countries with BSL-3 laboratories, with a notable concentration in the United States and China. Among these, only eight countries reported more than 10 unique institutions possessing BSL-3 capabilities [23]. It is crucial to recognize that while some laboratories may be colocated, others might exist that were not captured in this research.

The United States' prominence in BSL-3 infrastructure can be attributed to its stringent federal oversight of high-risk pathogen research. This oversight is often linked to federal grants, mandating publication of research findings, detailed methodological descriptions, and transparent reporting of safety measures [26, 27]. In contrast, China's substantial BSL-3 capacity likely stems from its prioritization of biotechnology research and development (R&D), with infectious diseases and public health identified as key governmental focuses. Strategic plans, such as the Medium and Long-Term Science and Technology Strategic Program (2006–2020) and the 13th Five-Year Science and Technology Innovation Plan, have driven significant investment in biotechnology-related infrastructure, including BSL-3 laboratories, research and innovation centers, and technical training facilities [28].

Given that the research was based on accounting for laboratories based on the number of scientific publications, it is to be expected that countries with strict regulatory supervision which encourage their researchers to publish the results of their research would be more representative, as highlighted in the CSET report. However, this also draws attention to the fact that if these structures had not been counted based on the number of scientific publications, countries with less rigorous supervision would likely have been able to count a greater number of laboratories, since they do not require more robust facilities with greater control, which could be more widespread.

**Figure 1:**
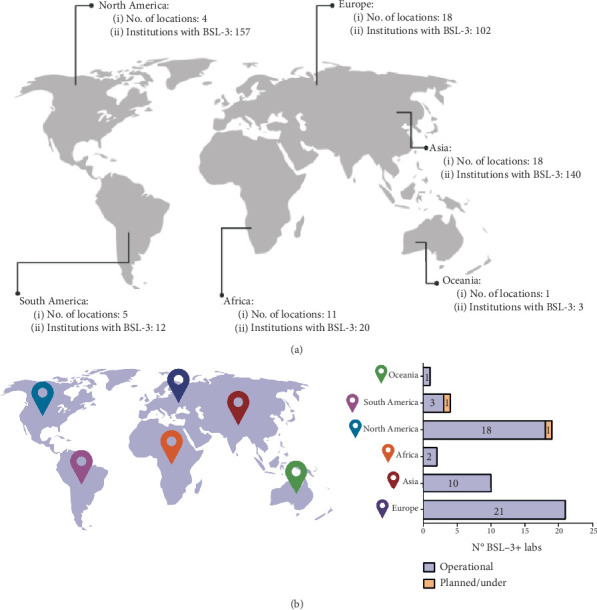
Identification of BSL-3 laboratories by continent: (a) according to CSET survey [23] and (b) according to Global BioLabs survey [29]. Created with BioRender.com.

Figure 1a,b illustrates the geographical distribution of BSL-3 laboratories and associated institutions, highlighting North America's dominance with 157 institutions, 148 of which are in the United States. Asia follows with 140 institutions, primarily concentrated in China. In South America, Brazil stands out with five identified BSL-3 institutions.

This investigation carried out by CSET was based on the search for publications carried out by the institutions with BSL-3 laboratories. Thus, it is essential to emphasize that this number may vary since institutions that do not publish their research or that do not appear in the database investigated may not have appeared in the results. To compare the data found and bring another perspective to BSL-3 laboratory identification worldwide, a screening carried out by Global BioLabs is presented. According to their screening, 57 laboratories are categorized as BSL-3+ (or plus/enhanced). BSL-3+ are laboratories that have adopted additional physical and operational biosafety measures to conduct higher-risk research activities (usually to operate with animal pathogens classified as 3 or 4 biological risks). According to this research, all these laboratories are active, except for a unit located in Brazil, which is being planned [29] (Figure 1).

Europe boasts the highest number of BSL-3 laboratories (*n* = 21), predominantly focused on animal pathogens like the H5N1 avian influenza virus. These laboratories are divided between public health and university research settings. Additionally, at least 69 BSL-4 laboratories, representing the highest level of biocontainment, were identified globally, with most dedicated to human health research [29].

Discrepancies between this survey and other reports can be attributed to variations in BSL-3 definitions, outdated or incomplete data, the inclusion of nondesignated BSL-3 facilities, and differing search methodologies [30]. Data on laboratories may be outdated or incomplete due to a lack of access to more in-depth information or the availability of real-time data. This may involve the relocation or closure of structures or a change in BSL over time. In addition, some mappings may include laboratories that have not been officially designated as BSL-3, because as BSL-3+ are a subgroup of BSL-3, some reports may or may not consider including them in their BSL-3 mappings. Finally, depending on the inclusion criteria and search methodology, some mappings may consist of only government laboratories, excluding, for example, existing research laboratories in universities or other centers or vice versa [23, 30]. It is also important to say that if there is no requirement to inform the government about research in BSL-3 laboratories, the information on the number of high-containment laboratories may end up not being as comprehensive. For example, in the United States, the reporting requirement is only for laboratories that are working with select agents (Select Agent Program) as discussed above; otherwise, there is no requirement [24].

In Brazil, the Ministry of Health initiated in 2002 a plan to implement a network of BSL-3 laboratories as part of a National Biosafety Policy to provide structural and operational conditions for laboratory diagnosis that would allow conditions at this level of biosafety. As a result of this plan, 12 laboratories distributed across the country's regions were delivered to work on bacteriology and virology with a focus on tuberculosis [16, 31]. However, like other developing countries, Brazil faces problems in its disease prevention and control structure due to the scarcity and uneven geographical distribution of facilities at this containment level [10]. When comparing the number of laboratories in Brazil mentioned in the previous paragraph with the number of laboratories identified in the CSET report, it is possible to see the discrepancy in the results since only five BSL-3 laboratories were identified in the country in the report. One explanation is that during the COVID-19 pandemic, these laboratories only worked on the molecular diagnosis of disease and stopped carrying out other research activities, which may have influenced the number of scientific publications, which was the parameter used by the CSET to track BSL-3 laboratories. These 12 laboratories belong to the Brazilian Ministry of Health and are about to health institutions, such as the Oswaldo Cruz Foundation (Fiocruz). Taking the laboratory located in the state of Bahia as an example, it was used exclusively for diagnostic purposes, as it is the only BSL-3 laboratory in this state and only returned to full operation for research use at the beginning of 2023 [31].

**Figure 2:**
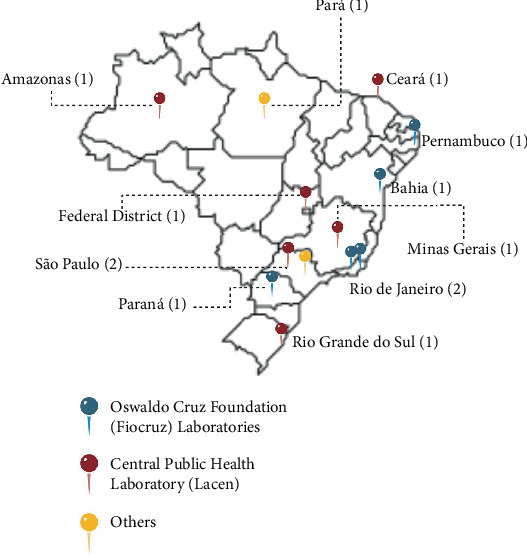
BSL-3 laboratories identified in Brazil per state (created with BioRender.com). *Source:* [21, 32].

There has not been a significant evolution in this figure over time. Recent data indicate the presence of 13 laboratories distributed throughout Brazil, as shown in Figure 2 [21, 32]. This discrepancy is because Brazil lacks standardized criteria and a legal framework for BSL-3 laboratories. Consequently, only two of these laboratories have undergone international certification, following FAO recommendations. It is worth noting that this number includes the so-called BSL-3Ag and ABSL-3 laboratories used in agriculture and animal experiments, respectively [21, 32]. This discrepancy highlights the need for comprehensive standards and regulations in Brazil to ensure the safe and consistent operation of BSL-3 facilities.

As previously mentioned, Brazil lacks a specific standard for BSL-3 laboratories. Therefore, foreign bases such as those from the United States, Canada, and Australia are often used when it comes to commissioning and certifying these laboratories. Alternatively, information from national documents is incorporated into the process. The primary normative references on which these federal documents are based for the determination of the general biosafety guidelines are the WHO Laboratory Biosafety Manual, the Organic Health Law (Law No. 8.080, September 19, 1990), the resolutions of the National Health Surveillance Agency (Anvisa), the Occupational Safety and Health Legislation (Law No. 6,514, December 22, 1977), the Regulatory Standards of the Ministry of Labor and Employment (Ordinance No. 3214, June 8, 1978) and the resolutions of the National Environment Council (CONAMA) [33]. By referencing these international and national documents, Brazil aims to establish a framework for biosafety practices and regulations for BSL-3 laboratories.

Despite the existence of international standards, developing specific, comprehensive standards for BSL-3 laboratories in the country would help ensure consistency and safety in operating such facilities. Since Brazil lacks this type of regulation, more standardized information is difficult because the country tends to rely on information extracted from different documents, which may not be entirely appropriate to the country's reality, in economic terms, for example. Furthermore, due to the different types of studies that can be carried out in these facilities, the recommendations can differ depending on the type of pathogen being worked on (human pathogens, animal pathogens, and agricultural pathogens) and the conditions that may be necessary for some may not apply to others. This ultimately reinforces the need for national standardization of this information according to the type of level or according to different standards for each type of application so that there is no confusion or misapplication when thinking about the construction and monitoring of a high containment laboratory, while also thinking about the economic and social reality of the country [34].

### 2.2. Pandemic COVID-19 Impacts BSL-3 Facility Investments

The COVID-19 pandemic underscored the critical role of robust local infrastructure, including diagnostic laboratories, hospital systems, and biosafety-level facilities—and pre-existing research networks in effectively responding to health emergencies, particularly in developing countries [10, 35]. While the construction of BSL-3 laboratories does not eliminate external dependencies, it enhances a nation's capacity to isolate and propagate pathogens, conduct diagnostics and R&D, and bolster public health preparedness, potentially reducing reliance on external resources, especially for neglected diseases. Many low- and middle-income countries faced significant limitations in BSL-3 laboratory availability. As a result, several institutions temporarily adapted BSL-2 laboratories to conduct SARS-CoV-2 diagnostic activities, despite not being ideally suited for handling high-concentration viral cultures. These adaptations, often guided by enhanced biosafety protocols, were aimed at mitigating urgent diagnostic gaps but also introduced increased biosafety risks and delays in confirmatory testing [36, 37, 38].

Being scientifically and infrastructurally prepared to address emergencies is of crucial importance for local public health maintenance. This can be exemplified by the case of Zika virus (BSL-2 or BSL-3 depending on the procedure), which was deemed the largest outbreak of an arbovirus in history. It peaked in 2015, causing a significant surge in cases in Brazil and rapidly spreading to other Latin American countries [39]. In this case, affected countries coordinated an internal response.

Important measures can be used to create an internal response to health emergencies and, for public health authorities to be able to use these measures effectively, timely and robust information must be shared between laboratories. This includes data on the microorganism's genome, prevalence, diagnostic test results, information on its behavior, and transmission dynamics. In more detail, surveillance and monitoring allow for the collection and analysis of data on the number of cases, geographical spread, and demographic information. Diagnostic tests allow cases to be followed up, tracking the progression of the disease. It is important to mention the importance of access to medical supplies for the treatment of cases, as well as public awareness of the disease and awareness of symptoms and how it is transmitted, in addition to ways in which the population can protect itself. In the specific case mentioned above, the Zika virus, another measure to be taken is data on the control of the vector, the *Aedes aegypti* mosquito, enabling the creation of strategies to prevent transmission. This data needs to be shared in collaboration between laboratories, public health agencies, and other interested parties, fostering the construction of effective responses [40–42].

This coordinated response was made possible due to investments in scientific research focusing on various arboviruses at both federal and state levels over the preceding decades. These investments provided a solid base for conducting studies on the Zika virus, allowing the national production of knowledge necessary for an efficient response in terms of public health [35].

Compared to the COVID-19 example, although clinical specimens (with or without suspected infection) can be handled for routine diagnosis in BSL-2 facilities, isolation and propagation of high concentrations of the virus can only be performed in BSL-3 facilities, at a minimum [43]. Not all countries have access to BSL-3 laboratories or other high-containment facilities required to handle situations like these.

Given the limited prior research on this virus, the pandemic has underscored the urgent need to establish and expand high-containment laboratories, particularly BSL-3 and BSL-4 facilities. These laboratories play a critical role in enabling safe pathogen handling, accelerating diagnostic development, and supporting vaccine and therapeutic research. Strengthening this global infrastructure is essential to ensure equitable preparedness for future health emergencies. They are essential for several reasons, including the possibility of isolating and propagating high-risk pathogens, as previously mentioned. These structures offer safe conditions for carrying out these activities and are essential for understanding the biology and pathogenesis of disease, as well as enabling the safe development of diagnostic methods and monitoring of emerging diseases, and can contribute to tracking the spread and safe processing of clinical samples.

In addition, these activities also contribute to the development of treatment and prevention methods, such as vaccines and other therapies, driving medical innovation and the response to pandemics. In general, the existence of BSL-3 laboratories allows a country to be better prepared with the necessary resources to develop these activities, reflecting stronger public health in the face of health threats [36, 37]. Simultaneously, there is a pressing requirement to allocate resources and provide training for human resources to enhance their preparedness and effectiveness in responding to high-risk emergency scenarios [10, 44].  Table 2 showcases examples of BSL-3 laboratories constructed or repurposed during the pandemic.

Besides pointing to the need for investment in high-level BSL, other impacts could be observed during and after the pandemic. There was an increased demand for research and clinical studies on infectious diseases, reinforcing the exploration of and investment in facilities that provide for the research and investigation of highly contagious viruses in a safe way, fostering this demand for these structures [47]. Furthermore, it is essential to mention that the need for sharing resources and knowledge between institutions encouraged international cooperation between the countries and their structures, accelerating research and contributing to a greater understanding of the virus. This cooperation has also perpetuated outside COVID-19 within the context of developing new diagnostics, treatments, and vaccines against other infectious diseases [48, 49].

Another significant impact has been the emphasis on the importance of strict safety regulations and guidelines regarding work with highly infectious agents. Governments and health organizations have acted constantly with updates, reinforcing the need for adherence to biosafety regulations and adequate investment in training for researchers working in high-level BSL, as well as for other professionals who worked on the front lines of the pandemic. This emphasis could be perpetuated and internalized in the face of the study of other diseases and care within healthcare environments [50, 51].

The global response to the pandemic underscored the critical importance of robust national preparedness for public health emergencies. This heightened awareness spurred significant investments in both the construction of new high-level biosafety facilities and the enhancement of existing laboratories. These upgrades focused on expanding operational capacities and aligning procedures and standards with the demands of pandemic response.

The urgent need for mass testing and rapid virus identification in the early stages of the COVID-19 pandemic led to the widespread temporary utilization of BSL-2 laboratories, which were enhanced to safely accommodate low concentrations of SARS-CoV-2 by WHO guidelines. This involved implementing additional safety measures such as increased respiratory protection, use of Level 3 PPE, and designated areas for donning and doffing PPE [13, 52]. This adaptation of BSL-2 laboratories—often referred to as BSL-2+ or BSL-2 enhanced—enabled a rapid scale-up of testing capacity. This was particularly important given the limited availability of BSL-3 and BSL-4 laboratories, which require advanced engineering controls, dedicated ventilation systems, and specialized training. By upgrading existing BSL-2 diagnostic laboratories and academic research facilities, many countries were able to expand their diagnostic capabilities and respond more effectively to the demands of the pandemic [53].

It is essential to mention that despite the lower level of biosafety, it does not mean that the protocols have been neglected. However, despite the benefits, these laboratories' rapid construction/adaptation has raised questions about the possible associated biological risks and perhaps the improper use of the facilities for investigations requiring higher BSLs [53]. With this in view, investment in building new BSLs was also observed as a priority in health care in different countries. Associated with this trend is the increase in funding for health research, reflecting greater awareness of the importance of investment in public health and infectious disease preparedness. This includes both research grants and targeted investments in infrastructure such as BSL-3 laboratories, genomic surveillance platforms, mobile diagnostic units, and biorepositories, all of which are essential for strengthening health systems and pandemic readiness [54, 55].

These structures allow their use in the face of new emergencies, since with the increased knowledge and training of countries, these investments can place developing countries as part of the front line in facing other outbreaks and pandemics. Thus, establishing federal and state regulations for the availability of funds for investment in health, more specifically in the fight against pandemics, is necessary as an initiative for governments and decision-making. In January 2021, the Group of 20 (G20), the leading forum for international economic cooperation, established the High-Level Independent Panel (HLIP). The HLIP proposes practical solutions to ensure consistent and sustainable funding for global initiatives to prevent, prepare for, and respond to pandemics, determining that the annual investment in pandemic preparedness should be 12% of gross domestic product (GDP), as a way of maintaining a specific financial fund for emergency health situations. For the COVID-19 pandemic, the support provided for health was 252% higher than that recommended by HLIP. In addition, estimates indicate that between 2022 and 2026, governments in 17 of 137 developing countries should increase national government health spending by an additional 1% of GDP [55, 56].

It is essential to analyze global health investments during the onset of the pandemic to understand the financial picture of this period and assess the proportion of change in spending needed to adequately prevent future global pandemics. Consequently, pandemic preparedness and response have become subjects of intense interest and debate. According to the WHO, pandemic preparedness involves having national response plans, adequate resources, and the capacity to support operations during a pandemic [57, 58]. All these factors are critical in promoting investments for the adequacy of countries in preparing for future outbreaks and/or pandemics.

## 3. Future Perspectives

### 3.1. New Health Emergencies and the Importance of BSL-3 Facilities' Investments

Since the existence of humanity, infectious agents have been responsible for causing high mortality rates, and, as the population increases and globalization has become increasingly tangible, contact with new or reemerging pathogens is more pronounced since, currently, their spread in previously unexplored environments is more facilitated, making them less confined to geographical or climatic boundaries, providing the emergence of situations that can affect public health globally [59]. Thus, technological and scientific advances must accompany this process to provide greater security to countries in the face of health situations, whether severe or not.

Given the historical precedent, it is indeed expected that pandemics will continue to occur. Pandemics are a natural consequence of our interconnected world, where diseases can rapidly spread across borders due to travel, trade, and other factors [60, 61]. Scientists warn of the emergence of new zoonoses in the future, mainly related to respiratory diseases, highlighting this risk as something of greater probability from now on. Large livestock farms, population growth, occupation of new natural areas, climate change, and the commercialization of wild animals are some factors that facilitate zoonotic events and directly affect the possibility of new infectious diseases [62].

Monitoring these factors can help prepare for emergency health situations. For example, weather monitoring can provide essential climate data on natural phenomena such as floods, hurricanes, or droughts, factors that directly influence new infectious agents' emergence. Warning about these phenomena can prepare the population and predict impacts on infrastructure and agriculture [63]. Monitoring air pollution and using the information obtained from this monitoring to develop means of controlling pollution caused by population growth and environmental exploitation are necessary to reduce stress on the ecosystem, improving overall ecological health, given that pollution is one favoring factor that favors the spread of respiratory diseases [64, 65].

Monitoring these and other factors can contribute to a better understanding of how they influence disease transmission patterns and inform public health strategies and interventions. It also highlights the need for preparedness in infrastructure planning, including investment in biosafety-level laboratories (e.g., BSL-3), mobile diagnostic units, and environmental surveillance systems that support advanced research and rapid response capabilities [66, 67].

Thus, the latest COVID-19 pandemic must be seen as a rehearsal for what may happen in the not-so-distant future, which may be even more damaging to human health [68]. Data estimated by Metabiota, a San Francisco–based company that tracks infectious disease risks and outbreaks, says that for the next 10 to 25 years, there is a 22%–28% and 47%–57% probability, respectively, of a new emergency like or of greater magnitude than COVID-19 occurring. These data are based on historical modeling suggesting an increase in the frequency and severity of outbreaks and epidemics caused by zoonosis, driven by human activities and their impact on the environment, according to the factors previously cited [69, 70].

Through this historical modeling, financial institutions can be assisted with external events and phenomena that may cause a health emergency. Relying on this predictive data is one of the strategies adopted by government agencies, development institutions, and private and public sector companies in making decisions on what to invest in for the coming years [71]. Within this context, despite existing predictions, more advanced studies will be found more frequently in the coming years.

WHO has published a first report named “Imagining the Future of Pandemics and Epidemics,” which seeks to explore future infectious threats in a short period (3–5 years). This study was commissioned by WHO in 2021 and carried out by Arup. This multinational company provides professional engineering, design, planning, project management, and consultancy services for all aspects of the built environment. The objective was to develop a set of scenarios for the future of the COVID-19 pandemic, as well as for other threats involving infectious agents. The methodology was based on the following morphological approach: (i) understanding the system of change, (ii) trend research and identification, (iii) key factor identification, (iv) morphological box and projections, (v) scenario development, and (vi) engagement and communication [72].

This research will be responsible for creating opportunities for reflection on future health emergencies, allowing the discussion of future scenarios and helping countries to prepare to deal with new or re-emerging infectious diseases. Today, pandemic preparedness must be shared globally, a commitment that must be increasingly established between governments, scientists, the community, public and private sector authorities, the media, and the population itself [73]. In the future, nations should share more information about studies, ensuring that the benefits of technological innovation are widespread so that health systems are better prepared, assisted by solid scientific bases, and adequate financial provision for investment in structures and knowledge. This effort will be responsible for helping to recognize threats, establish trust, and collect the best preparation for future eventualities, especially when it comes to assisting developing countries in scientific and technological development [74, 75].

Over the years, BSL-3 laboratories have evolved in terms of their containment architecture, engineering controls (such as HVAC and HEPA filtration systems), biosafety policies, and skilled human resources. These improvements have enabled safer work with moderate- to high-risk pathogens in controlled environments. In light of the ongoing threat of emerging infectious diseases, the global demand for such facilities has intensified, as they are crucial for supporting public health infrastructure and pandemic readiness [38].

Investment in these high-containment facilities is closely tied to national development, as the design, construction, and maintenance of BSL-3 laboratories require substantial financial resources. These include operational expenditures for biosafety supplies, continuous personnel training, and sustained R&D. Despite high costs, such investments have enabled countries to proactively identify and categorize emerging pathogens [76, 77]. Given the financial disparities across nations, international efforts are increasingly focused on knowledge transfer and capacity-building to support global health security [78].

As far as investment in high-containment laboratories is concerned, some trends are expected in the future. The primary trend is increased investment in research, preparedness, and the creation of government initiatives for emergency support. These health emergencies have significantly highlighted the importance of investing in this type of structure to develop more effective vaccines, therapies, and diagnostics in the future [79]. In terms of initiatives to better prepare countries for future pandemics, the WHO's Preparedness and Resilience for Emerging Threats Initiative (PRET) is aimed at guiding integrated planning in response to respiratory pathogens by incorporating new tools and approaches to disseminate knowledge collectively. Through PRET, the organization will use an integrated system considering the common knowledge between groups of pathogens, promoting technical guidance and support to strengthen emergency preparedness and response. It is worth noting that despite the concern about pathogens that can cause respiratory emergencies, arboviruses are another group of pathogens considered a priority for country preparedness [78].

Another trend identified is the extension of the capacity of existing laboratories around the world to meet the need to update in line with the latest safety standards. As previously mentioned, since there are no international standards dedicated to the installation of BSL-3 or BSL-4 laboratories, standards such as ISO/IEC 17025:2017 are one of the WHO-recognized standards that guide quality assurance for the implementation of laboratories with a medical or environmental purpose. As such, it is expected that existing laboratories will seek to update their processes as a way of ensuring that they are compliant and prepared to carry out research involving high-risk pathogens [80].

### 3.2. Preparing Countries for New Emergencies: What to Expect From an Economic Point of View

Investing in high-containment laboratories and strengthening national capacity to respond to health emergencies are essential for ensuring the safety and well-being of global populations. While recent years have seen an increase in such investments, there remains a need for strategic evaluation of funding priorities and resource allocation. Beyond physical infrastructure, critical areas for sustained investment include advanced technological platforms, R&D, workforce training, real-time surveillance and monitoring systems, vaccine and therapeutic stockpiling, risk communication infrastructure, and the integration of public health networks across sectors. These components should be aligned under coordinated frameworks such as the One Health approach and international cooperation initiatives, ensuring that countries—especially those with limited resources—can prepare for and effectively manage future infectious disease threats. Clear investment priorities, guided by global best practices and local needs assessments, are necessary to transition from reactive responses to proactive health security strategies [81].

Countries are constantly working to develop plans, such as the National Action Plans for Health Security (NAPHS), a country-owned, multiyear plan that can accelerate the implementation of International Health Regulations (IHR) by focusing on what is a priority for health security, identifying global partners, and allocating resources for the development of health security in member countries [82]. However, raising full funding for these activities has been a difficult task [83, 84]. WHO researchers investigated studies estimating the costs of improving and preparing countries against emergency health situations published between January 1, 2000, and May 14, 2021, by searching different databases [85]. Some inclusion criteria were adopted: preparation costs at the national and/or global level in at least 10 countries, covering two or more technical benchmarks according to WHO Benchmarks for IHR Capacities [86].

The findings indicated 10 studies that met these inclusion criteria, with varying calculation methods. Four of the publications originated from peer-reviewed academic journals, while six were reports from grey literature. Most of these studies were conducted by international organizations or authors affiliated with institutions in high-income nations, which consistently invest a greater share of their GDP in R&D. For instance, the United States and United Kingdom allocate over 2.5% of their GDP to R&D, according to OECD [87]. However, there were a couple of exceptions: one study involved three authors primarily linked to organizations in Brazil, China, and Kenya [88], and another study was authored by someone primarily affiliated with an organization in Liberia [89]. The cost estimates found ranged from $1.6 billion a year invested in 139 low- and middle-income countries to $43 billion a year over the next 10 years, applied to support different countries and to global support in terms of helping to implement initiatives, such as R&D focused on the development of new vaccines, therapies, and diagnostics. Overall, these recent estimates indicate that the costs of better preparedness against new health emergencies amount to more than $31.1 billion per year [85].

The study confirmed that investment costs tend to be higher in low-income countries, primarily due to the greater need for baseline capacity strengthening. This includes capital investments in constructing or upgrading high-containment laboratories (e.g., BSL-3), establishing appropriate engineering controls, and building a trained biosafety workforce. These findings underscore the importance of targeted international support mechanisms, including public–private partnerships and coordinated funding frameworks, to reduce disparities and promote equitable global preparedness [89]. It is important to point out that the reliability of these studies is difficult to assess since many studies report summary and noncomprehensive information. Given that needs can differ depending on the country's level of development, the estimated investment costs can be different. As such, the cost estimates reported should be used as a basis for predictions for each country, based on national needs, not disregarding the unique priority of preparing these countries against emergency health situations [90]. Future initiatives could include in their analysis an emphasis on assessing quality or identifying possible biases in the search for this data.

Looking from another perspective, benefits, and even financial returns can be achieved by investing in universal health coverage (public health), protecting people from health emergencies, and supporting healthy populations. Estimates state that, in terms of universal coverage, these investments could contribute to saving up to 24.4 million lives (period 2019–2023), bringing a financial return of around $1.4 for every dollar invested, which would increase to $9 for every dollar invested if the inherent value of human life is considered [91]. This is because conditions such as mental health, which, as observed during the COVID-19 pandemic, cases of depression and anxiety increased during this period and continue to be a symptom to this day, can be better treated with the appropriate financial support and preparation for health emergencies [92]. In addition, economic and social benefits because of avoided deaths and disabilities can be guaranteed by investing in vaccination programs, especially for low- and middle-income countries [93].

A case study showed that based on a 25% probability of the emergence of a new pandemic comparable to COVID-19 in the next decade, the probability-adjusted benefit of preventing this new health emergency would be $2.5 billion [94]. Therefore, the benefits of better control and management of an emergency, once it has arisen, can be estimated by comparing the countries with the best and worst performance in responding to the COVID-19 pandemic, with probability-adjusted benefits of $750 billion in the first year and $1.2 trillion over 3 years [95, 96]. Therefore, investment in this objective of preparing countries for new health emergencies is not just based on spending but includes generating profits based on impactful results in terms of protecting public health and other long-term benefits [96].

### 3.3. Regulatory Issues and New Technologies in Brazil: What Are Expected?

In Brazil, the concept of biosafety was formally established by Law No. 8974 (1995) and further defined in Law No. 11,105 (2005), known as the Biosafety Law, which focuses on the regulation of genetically modified organisms (GMOs) [97, 98]. This legislation assigns responsibilities across federal ministries, including Health and Agriculture, and led to the creation of the National Technical Commission on Biosafety (CTNBio) under the Ministry of Science, Technology, and Innovation. CTNBio oversees research involving GMOs. In parallel, pathogen classification systems in Brazil follow models like those adopted in the United States [98].

While biosafety is recognized as a strategic area, comprehensive national regulations are lacking for non-GMO biological agents and for high-containment laboratories such as those classified as BSL-3. In the absence of specific national guidelines, the commissioning and certification of such facilities often rely on international standards and frameworks [33]. As a result, Brazilian biosafety practices draw from various legal instruments—many of which were not designed for high-containment environments—leading to regulatory fragmentation [99].

This regulatory dispersion refers to the fragmentation of biosafety-related responsibilities across multiple ministries and agencies, such as Health, Agriculture, Defense, and Science and Technology. This leads to overlapping mandates, inconsistent implementation of biosafety norms, and limited coordination among institutions. Although Brazil has a solid track record in biomedical research, its experience in managing high-risk pathogens under strict biocontainment conditions is still developing. To address these regulatory challenges, the implementation of a national biosafety authority with centralized oversight—as well as a unified pathogen inventory and registration system—could contribute to harmonizing standards, strengthening preparedness, and aligning with international best practices [100]. Building and maintaining BSL-3 facilities involve substantial investment in high-containment architecture, HVAC systems, HEPA filtration, autoclaves, and secure waste management infrastructure, as well as the recruitment and continuous training of specialized personnel [101]. The availability of trained biosafety professionals is also a concern, particularly regarding technical knowledge that is essential to safeguard both public health and the environment [33].

To address these challenges, the Brazilian federal government is currently developing the National Biosafety and Biosecurity Policy (PNBB), led by the Ministry of Defense in collaboration with other ministries and agencies, including Anvisa, ABIN, the Ministry of Health, and CTNBio [33]. The PNBB is aimed at providing integrated and updated national guidelines covering both GMOs and non-GMO biological agents. In addition to strengthening internal coordination and risk management, the policy seeks alignment with international frameworks—such as the IHR—to enhance Brazil's capacity for responding to public health emergencies; foster responsible scientific innovation; and ensure the protection of human, animal, and environmental health within the country's specific scientific, institutional, and economic realities [112].

One notable milestone in this effort is the planned implementation of Brazil's first maximum biocontainment facility (BSL-4), known as Orion. Scheduled for completion by 2026, Orion will be the first laboratory of its kind in Latin America and will be colocated with the Sirius synchrotron light source. Together, these facilities are aimed at providing cutting-edge capabilities for studying high-risk microorganisms and accelerating biomedical research [102]. For instance, due to a lack of domestic infrastructure, samples of the Sabia virus—an arenavirus responsible for Brazilian hemorrhagic fever—have historically been stored abroad, limiting research efforts within Brazil [103, 104].

In tandem with the Orion project, the Ministry of Health issued Ordinance No. 1736 in November 2023, establishing a working group to define governance models, regulatory mechanisms, and monitoring frameworks for BSL-4 operations in the country [105]. These initiatives also align with Brazil's commitments under international frameworks such as the IHR, which guide disease surveillance and laboratory biosafety on a global scale [33, 106].

The implementation of a BSL-4 facility is expected to significantly strengthen Brazil's capacity to detect, contain, and study emerging and reemerging high-risk pathogens, while complementing and expanding the existing national BSL-3 network. This advanced infrastructure will enhance not only pathogen research capabilities but also preparedness and response mechanisms aligned with international biosafety and biosecurity standards [107]. Continued investment in regulatory harmonization and biosafety capacity building is likely to enhance Brazil's readiness to respond to future infectious disease threats and support the development of new diagnostics, therapies, and vaccines.

## 4. Conclusions

Even in the current era of advanced immunization strategies, future threats from infectious diseases remain a significant global concern. The COVID-19 pandemic exposed gaps in emergency preparedness, highlighting the urgent need for continuous investment in robust, high-containment infrastructure capable of supporting rapid diagnostic, containment, and research efforts. This includes the construction and maintenance of BSL-3 and BSL-4 laboratories, as well as the expansion of genomic surveillance platforms and mobile diagnostic units.

High-income countries such as the United States and China have demonstrated leadership during public health crises, largely due to their well-established research ecosystems, consistent R&D investment, strategic incentive programs, and strong regulatory oversight. However, effective response to health threats is not exclusively dependent on economic status. Strengthening local laboratory capacity, fostering interconnected research networks, and expanding workforce training are essential for any country to respond effectively. To reduce global disparities in pandemic readiness, international cooperation and the dissemination of biosafety and biosecurity expertise must be prioritized. Initiatives that support low- and middle-income countries through knowledge transfer, technical support, and sustainable financing models are critical to building a more resilient and equitable global health infrastructure. Investment in preparing for emergencies is essential not only to ensure national security but also to strengthen the country's ability to respond to public health threats. In Brazil, the government has made progress in biosafety, particularly in response to biotechnological developments. However, challenges remain due to regulatory dispersion—understood as the fragmentation of responsibilities across multiple ministries and agencies—which results in overlapping mandates and inconsistent implementation of safety standards. The PNBB seeks to address these challenges by consolidating existing norms, fostering interagency coordination, and potentially establishing centralized mechanisms such as a unified pathogen inventory system. These measures are aimed at promoting national scientific and technological development while ensuring the protection of human, animal, plant, and environmental health.

Therefore, nations should continue to improve their capacity to respond to health emergencies, focusing on international initiatives and coordination to enhance compliance with essential requirements, while implementing preventive measures against emerging infectious diseases and reemerging. These approaches will serve to better equip countries to adapt to the changing global scenario and the possibility of future health emergencies related to infectious diseases, always seeking to update and invest in new sophisticated technologies for the scientific and technological advancement of the country.

In this context, strengthening international coordination for biosafety standards, supporting public–private investment models for the construction and maintenance of BSL-3 laboratories, and promoting integration with One Health surveillance systems are key strategies. These actions can enhance global preparedness, ensure equitable access to critical containment infrastructure, and support timely response to future public health threats.

## Figures and Tables

**Table 1:** Requirements for the installation of a BSL-3 laboratory.

**Aspect**	**Requirements**
Physical structure	The structure must have sufficient space for the allocation of equipment and the safe development of activities
	The primary access must have a structure compatible with a BSL-2 laboratory
	Double-door antechamber that defines access flow and must be self-closing
	Controlled, restricted, and secure access to the BSL-3 area
	Exclusive technical floor
	Exclusive engineering facilities and systems
	Dedicated and dedicated emergency power system
	Voice and data communication system

Air-conditioning and exhaust system	Specific, exclusive, and independent air-conditioning system for the BSL-3 area
	The air-conditioning system must ensure the parameters of the differential pressure gradients and airflow
	The airflow in the facility should always be in a single direction, that is, from the least contaminated area to the most contaminated area
	Alarm for airflow monitoring to alert laboratory and engineering staff of any control set parameters
	Extract air may not be recirculated within the room or to any other area of the building
	Filtration through HEPA^a^ filters
	Inflation and exhaust systems should be designed to maintain operational balance and perfect compatibility with BSC

Equipment and internal arrangements	Class II BSC is available inside the BSL-3 area
	Barrier autoclave provided inside the laboratory
	Specific washing/expurgation area
	Effluent treatment system
	Emergency showers and eye wash were provided at the exit sluice
	Sink, if existent, with hands-free tap
	Windows should be fixed and sealed
	Penetrations and finishes in the BSL-3 area should be sealed
	Luminaires, diffusers, and roof grilles should have their joints sealed to control air leakage from the containment environment
	Doors should allow for tightness during biological decontamination processes
	Benches and chairs should be waterproof and resistant to disinfectants, acids, alkalis, organic solvents, and moderate heat
	Furniture and equipment must allow for decontamination
	The finished floor should be impervious to liquids, with sealed seams, resistant to chemicals, and have a surface that reduces the danger of slipping

^a^HEPA (high-efficiency particulate air): technology used in air filters with high efficiency in particle separation. In controlled environments, it is important for keeping the air free from contamination and preventing the spread of pathogens.

**Table 2:** Examples of BSL-3 laboratories adapted or created to support the fight against COVID-19.

**Laboratory**	**Country**	**Information**	**Reference**
Central Reference Laboratory (National Reference BSL-3 Laboratory)	Kazakhstan	It was set up before the pandemic and was the first laboratory in Central Asia accredited according to the new international standard for managing biological risks in laboratories. During the pandemic, it supported the development of vaccines, diagnosis, and isolation of SARS-CoV-2 variants.	[38]
MRIGlobal BSL-3	United States	Laboratories are managed by MRIGlobal, a nonprofit institution that aims to discover solutions for public and private scientific organizations. It was one of the first US institutions to dedicate one of its BSL-3 units to SARS-CoV-2 research.	[38]
Taiwan CDC BSL-3 Laboratory	Taiwan	Infrastructure previously dedicated to handling viruses, such as the Japanese encephalitis virus, was directed during the COVID-19 pandemic to diagnose SARS-CoV-2, contributing directly to the fight against the pandemic in the country.	[38]
Mobile Virology Research and Diagnostic Laboratory (MVRDL)	India	Created at the beginning of the pandemic, the MVRDL is characterized by being a mobile unit and, consequently, having greater flexibility. Its infrastructure was set up by WHO and Indian Council of Medical Research guidelines within a 15-day interval. The activities at the MVRDL are focused on research and diagnosis of SARS-CoV-2.	[45]
BSL-3 laboratories at Arizona State University	United States	Adaptation of the pre-existing BSL-3 infrastructure for handling SARS-CoV-2 by NIH and CDC guidelines. The new BSL-3 laboratory was used for COVID-19 diagnosis and research.	[46]

## Data Availability

Data sharing is not applicable to this article as no datasets were generated or analyzed during the current study.

## References

[1] Varotsos C. A., Krapivin V. F. (2020). A New Model for the Spread of COVID-19 and the Improvement of Safety. *Safety Science*.

[2] Bedford J., Farrar J., Ihekweazu C., Kang G., Koopmans M., Nkengasong J. (2019). A New Twenty-First Century Science for Effective Epidemic Response. *Nature*.

[3] Bayot M. L., King K. C. (2018). *Biohazard Levels*.

[4] Richmond J. Y., McKinney R. W. (2009). *Biosafety in Microbiological and Biomedical Laboratories*.

[5] Keckler M. S., Anderson K., McAllister S., Rasheed J. K., Noble-Wang J. (2019). Development and Implementation of Evidence-Based Laboratory Safety Management Tools for a Public Health Laboratory. *Safety Science*.

[6] Munson E., Bowles E. J., Dern R. (2018). Laboratory Focus on Improving the Culture of Biosafety: Statewide Risk Assessment of Clinical Laboratories That Process Specimens for Microbiologic Analysis. *Journal of Clinical Microbiology*.

[7] Joseph T. (2021). Management System Approach for Addressing Biosafety and Biosecurity of Emerging Pathogens in a Biosafety Level-3 Core Facility. *Applied Biosafety*.

[8] Mehmood W., Fareed M., Mohd-Rashid R., Ashraf M. U., Aman-Ullah A. (2023). The Role of Facilities Management in Fighting COVID-19 Outbreak: Evidence From Malaysian Public Hospitals. *Frontiers in Psychology*.

[9] Williams B. A., Jones C. H., Welch V., True J. M. (2023). Outlook of Pandemic Preparedness in a Post-COVID-19 World. *Npj Vaccines*.

[10] Souza T. M. L., Morel C. M. (2021). The COVID-19 Pandemics and the Relevance of Biosafety Facilities for Metagenomics Surveillance, Structured Disease Prevention and Control. *Biosafety and Health*.

[11] Noor R., Maniha S. M. (2020). A Brief Outline of Respiratory Viral Disease Outbreaks: 1889–Till Date on the Public Health Perspectives. *Virusdisease*.

[12] Cascini F., Hoxhaj I., Zaçe D. (2020). How Health Systems Approached Respiratory Viral Pandemics Over Time: A Systematic Review. *BMJ Global Health*.

[13] World Health Organization (2020). *Laboratory Biosafety Manual*.

[14] National Institutes of Health (NIH) Design Requirements Manual (DRM). https://orf.od.nih.gov/TechnicalResources/Documents/DRM/DRM2.108022024.pdf.

[15] World Organization for Animal Health (OIE) (2021). Terrestrial Animal Health Code, World Organization for Animal Health, United States. https://sont.woah.org/portal/tool?le=en.

[16] Mafra C. (2020). *Thinking a National Strategic Infrastructure: The Brazilian NB-4 Laboratory*.

[17] Jonsson C. B., Cole K. S., Roy C. J., Perlin D. S., Byrne G. (2013). Challenges and Practices in Building and Implementing Biosafety and Biosecurity Programs to Enable Basic and Translational Research With Select Agents. *Journal of Bioterrorism & Biodefense*.

[18] Beeckman D. S., Rüdelsheim P. (2020). Biosafety and Biosecurity in Containment: a Regulatory Overview. *Frontiers in Bioengineering and Biotechnology*.

[19] Kingsbury N., Bowser A., Depaoli G. (2009). *High-Containment Laboratories: National Strategy for Oversight is Needed*.

[20] National Science Advisory Board for Biosecurity (2011). Guidance for Enhancing Personnel Reliability and Strengthening the Culture of Responsibility. https://osp.od.nih.gov/wp-content/uploads/NSABB_WG_Draft_Report_on_Personnel_Reliability_and_Culture_of_Responsibility.pdf.

[21] Ministry of Health (2015). Biocontainment: The Management of Risk in High Biological Containment Environments BSL3 and BSLA3. https://www.gov.br/saude/pt-br/acesso-a-informacao/acoes-e-programas/sislab/publicacoes/sislab_15-0043-biocontencao.pdf.

[22] Sun D., Wu L., Fan G. (2021). Laboratory Information Management System for Biosafety Laboratory: Safety and Efficiency. *Journal of Biosafety and Biosecurity*.

[23] Schuerger C., Abdulla S., Puglisi A. (2022). *Mapping biosafety level-3 laboratories by publications*.

[24] Federal Select Agent Program (2022). Federal Select Agent Program Inspection Report Processing Annual Summary. https://www.selectagents.gov/resources/publications/docs/FSAP_Annual_Report_2022_508.pdf.

[25] National Institute of Health (NIH) (2024). *NIH Guidelines for Research Involving Recombinant or Synthetic Nucleic Acid Molecules (NIH Guidelines)*.

[26] National Institutes of Health (NIH) https://publicaccess.nih.gov/policy.htm.

[27] Office of Science and Technology Policy Public Access Memorandum. https://bidenwhitehouse.archives.gov/wp-content/uploads/2022/08/08-2022-OSTP-Public-Access-Memo.pdf.

[28] Center for Security and Emerging Technology, 2019 https://cset.georgetown.edu/wp-content/uploads/t0229_2019_statistical_communique_EN.pdf.

[29] Global BioLabs Global BioLabs Report 2023. https://static1.squarespace.com/static/62fa334a3a6fe8320f5dcf7e/t/6412d3120ee69a4f4efbec1f/1678955285754/KCL0680_BioLabs+Report_Digital.pdf.

[30] Johnson B., Casagrande R. (2016). Comparison of International Guidance for Biosafety Regarding Work Conducted at Biosafety Level 3 (BSL-3) and Gain-of-Function (GOF) Experiments. *Applied Biosafety*.

[31] https://www.bahia.fiocruz.br/laboratorio-de-nivel-de-biosseguranca-3-da-fiocruz-bahia-retorna-ao-funcionamento-pleno/.

[32] https://www.gov.br/saude/pt-br/acesso-a-informacao/acoes-e-programas/sislab.

[34] Maehira Y., Spencer R. C. (2019). Harmonization of Biosafety and Biosecurity Standards for High-Containment Facilities in Low- and Middle-Income Countries: An Approach From the Perspective of Occupational Safety and Health. *Frontiers in Public Health*.

[35] Vasconcellos A. G., Fonseca e Fonseca B. D., Morel C. M. (2018). Revisiting the Concept of Innovative Developing Countries (IDCs) for Its Relevance to Health Innovation and Neglected Tropical Diseases and for the Prevention and Control of Epidemics. *PLoS Neglected Tropical Diseases*.

[36] Filip R., Gheorghita Puscaselu R., Anchidin-Norocel L., Dimian M., Savage W. K. (2022). Global Challenges to Public Health Care Systems During the COVID-19 Pandemic: A Review of Pandemic Measures and Problems. *Journal of Personalized Medicine*.

[37] Cook N. L., Lauer M. S. (2021). Biomedical Research COVID-19 Impact Assessment: Lessons Learned and Compelling Needs. *NAM Perspectives*.

[38] Yeh K. B., Tabynov K., Parekh F. K. (2021). Significance of High-Containment Biological Laboratories Performing Work During the COVID-19 Pandemic: Biosafety Level-3 and -4 Labs. *Frontiers in Bioengineering and Biotechnology*.

[39] Mlakar J., Korva M., Tul N. (2016). Zika Virus Associated With Microcephaly. *New England Journal of Medicine*.

[40] Tumpey A. J., Daigle D., Nowak G., Rasmussen S., Goodman R. (2019). *The CDC Field Epidemiology Manual*.

[41] Haldane V., Jung A.-S., De Foo C. (2021). Strengthening the Basics: Public Health Responses to Prevent the Next Pandemic. *BMJ*.

[42] Nicholson A., Reeve Snair M., Rapporteurs J. H. (2016). *Global Health Risk Framework*.

[43] Centers for Disease Control and Prevention (CDC) Interim Laboratory Biosafety Guidelines for Handling and Processing Specimens Associated With Coronavirus Disease 2019 (COVID-19). https://www.cdc.gov/coronavirus/2019-ncov/lab/lab-biosafety-guidelines.html.

[44] Huang Y., Huang J., Xia H., Shi Y., Ma H., Yuan Z. (2019). Networking for Training Level 3/4 Biosafety Laboratory Staff. *Journal of Biosafety and Biosecurity*.

[45] Rao Y. S. (2020). Mobile Virology Research and Diagnostic Laboratory (MVRDL: BSL-3) for COVID-19 Screening, Virus Culturing and Vaccine Development. *Transactions of the Indian National Academy of Engineering*.

[46] Gillum D. R., Rice A. D., Mendoza I. A. (2022). The COVID-19 Pandemic Response: Biosafety Perspectives From a Large Research and Teaching Institution. *Applied Biosafety*.

[47] Park J. J. H., Mogg R., Smith G. E. (2021). How COVID-19 Has Fundamentally Changed Clinical Research in Global Health. *Lancet Global Health*.

[48] Coulibaly B. S. Rebooting Global Cooperation Is Imperative to Successfully Navigate the Multitude of Shocks Facing the Global Economy. https://www.brookings.edu/articles/rebooting-global-cooperation-is-imperative-to-successfully-navigate-the-multitude-of-shocks-facing-the-global-economy/.

[49] Brown G., Susskind D. (2020). International Cooperation During the COVID-19 Pandemic. *Oxford Review of Economic Policy*.

[50] Igoe K. J. How COVID-19 Has Changed the Standards of Worker Safety and Health — and How Organizations Can Adapt. https://www.hsph.harvard.edu/ecpe/how-covid-19-changed-worker-safety-and-health/.

[51] Occupational Safety and Health Act (OSHA) Guidance on Preparing Workplaces for COVID-19. https://www.osha.gov/sites/default/files/publications/OSHA3990.pdf.

[52] World Health Organization (WHO) (2020). *Laboratory Biosafety Guidance Related to Coronavirus Disease (COVID-19): Interim Guidance*.

[53] Yuan D., Gao W., Liang S., Yang S., Jia P. (2020). Biosafety Threats of the Rapidly Established Labs for SARS-CoV-2 Tests in China. *Environment International*.

[54] Rosa M. F. F., da Silva E. N., Pacheco C. (2021). Direct From the COVID-19 Crisis: Research and Innovation Sparks in Brazil. *Health Research Policy and Systems*.

[55] Global Burden of Disease 2021 Health Financing Collaborator Network (2023). Global Investments in Pandemic Preparedness and COVID-19: Development Assistance and Domestic Spending on Health Between 1990 and 2026. *Lancet Global Health*.

[56] G20 HLIP Report of the G20 High Level Independent Panel on Financing the Global Commons for Pandemic Preparedness and Response. https://www.mef.gov.it/en/ufficio-stampa/comunicati/2021/The-G20-establishes-a-High-Level-Independent-Panel-on-financing-the-Global-Commons-for-Pandemic-Preparedness-and-Response/.

[57] COVID-19 National Preparedness Collaborators (2022). Pandemic Preparedness and Covid-19: an Exploratory Analysis of Infection and Fatality Rates, and Contextual Factors Associated with Preparedness in 177 Countries, from Jan 1, 2020, to Sept 30, 2021. *Lancet*.

[58] World Health Organization (WHO) https://www.who.int/westernpacific/activities/preparing-for-pandemics.

[59] Peters A. (2018). The Global Proliferation of High-Containment Biological Laboratories: Understanding the Phenomenon and its Implications. *Rev Sci Tech*.

[60] Zhang L., Hu S., Xu G. (2023). Pandemic Uncertainty and Firm Investments. *Finance Research Letters*.

[61] Agarwal R., Gopinath G. (2022). Seven Finance and Trade Lessons From Covid-19 for Future Pandemics. *Oxford Review of Economic Policy*.

[62] Smith J. Q&A: Future Pandemics Are Inevitable, but We Can Reduce the Risk. https://ec.europa.eu/research-and-innovation/en/horizon-magazine/qa-future-pandemics-are-inevitable-we-can-reduce-risk.

[63] McClymont H., Hu W. (2021). Weather Variability and COVID-19 Transmission: A Review of Recent Research. *International Journal of Environmental Research and Public Health*.

[64] Lou J., Wu Y., Liu P., Kota S. H., Huang L. (2019). Health Effects of Climate Change Through Temperature and Air Pollution. *Current Pollution Reports*.

[65] Bontempi E. (2022). A Global Assessment of COVID-19 Diffusion Based on a Single Indicator: Some Considerations About Air Pollution and COVID-19 Spread. *Environmental Research*.

[66] Mayer J. D., Lewis N. D. (2020). An Inevitable Pandemic: Geographic Insights Into the COVID-19 Global Health Emergency. *Eurasian Geography and Economics*.

[67] Tong M., Hansen A., Hanson-Easey S. (2015). Infectious Diseases, Urbanization and Climate Change: Challenges in Future China. *International Journal of Environmental Research and Public Health*.

[68] G20 High Level Independent Panel A Global Deal for Our Pandemic Age. 2023. Report of the G20 High Level Independent Panel on Financing the Global Commons for Pandemic Preparedness and Response. https://pandemic-financing.org/files/wp-content/uploads/2021/07/g20-hlip-report.pdf.

[69] Agyarko R., Jamison D., Oppenheim B., Stefan C., Stephenson N. What’s Next? Predicting the Frequency and Scale of Future Pandemics. https://www.cgdev.org/event/whats-next-predicting-frequency-and-scale-future-pandemics.

[70] Cheney C. (2021). How might probability inform policy on pandemics? Metabiota has ideas. https://www.devex.com/news/how-might-probability-inform-policy-on-pandemics-metabiota-has-ideas-100427.

[71] Valera E., Jankelow A., Lim J. (2021). COVID-19 Point-of-Care Diagnostics: Present and Future. *ACS Nano*.

[72] World Health Organization (WHO) (2022). *Imagining the Future of Pandemics and Epidemics: A 2022 Perspective*.

[73] Colman E., Wanat M., Goossens H., Tonkin-Crine S., Anthierens S. (2021). Following the Science? Views From Scientists on Government Advisory Boards During the COVID-19 Pandemic: A Qualitative Interview Study in Five European Countries. *BMJ Global Health*.

[74] Bai X., Liu Y. (2016). International Collaboration Patterns and Effecting Factors of Emerging Technologies. *PLoS One*.

[75] Kazemian S., Fuller S., Algara C. (2021). The Role of Race and Scientific Trust on Support for COVID-19 Social Distancing Measures in the United States. *PLoS One*.

[76] Michelotti J., Yeh K., Beckham T. (2018). The Convergence of High-Consequence Livestock and Human Pathogen Research and Development: A Paradox of Zoonotic Disease. *Tropical Medicine and Infectious Disease*.

[77] National Research Council (2012). *Biosecurity Challenges of the Global Expansion of High-Containment Biological Laboratories*.

[78] World Health Organization (WHO) (2023). Preparedness and Resilience for Emerging Threats (PRET).

[79] Dagliati A., Malovini A., Tibollo V., Bellazzi R. (2021). Health Informatics and EHR to Support Clinical Research in the COVID-19 Pandemic: An Overview. *Briefings in Bioinformatics*.

[80] Hou M., Song D. L., Shi Z. L., Yuan Z. M. (2019). Quality Management in a High-Containment Laboratory. *Journal of Biosafety and Biosecurity*.

[81] Collins T., Akselrod S., Bloomfield A., Gamkrelidze A., Jakab Z., Placella E. (2020). Rethinking the COVID-19 Pandemic: Back to Public Health. *Annals of Global Health*.

[82] World Health Organization (WHO) (2022). *World Health Organization Strategy (2022-2026) for National Action Plan for Health Security*.

[83] Center for Strategic & International Studies (CSIS) (2019). *Ending the Cycle of Crisis and Complacency in U.S. Global Health Security*.

[84] Centers for Disease Control and Prevention (CDC) Joint External Evaluations (JEE) for Improved Health Security. https://archive.cdc.gov/#/details?url=https://www.cdc.gov/globalhealth/security/ghsareport/2018/jee.html.

[85] Clarke L., Patouillard E., Mirelman A. J., Ho Z. J. M., Edejer T. T.-T., Kandel N. (2022). The Costs of Improving Health Emergency Preparedness: A Systematic Review and Analysis of Multi-Country Studies. *EClinicalMedicine*.

[86] World Health Organization (WHO) (2019). *WHO Benchmarks for International Health Regulations (IHR) Capacities*.

[87] OECD Gross Domestic Spending on R&D. https://www.oecd.org/en/data/indicators/gross-domestic-spending-on-r-d.html?oecdcontrol-8027380c62-var3=2022.

[88] Dobson A. P., Pimm S. L., Hannah L. (2020). Ecology and Economics for Pandemic Prevention. *Science*.

[89] Peters D. H., Hanssen O., Gutierrez J., Abrahams J., Nyenswah T. (2019). Financing Common Goods for Health: Core Government Functions in Health Emergency and Disaster Risk Management. *Health Systems & Reform*.

[90] Yamey G., Jamison D., Hanssen O., Soucat A. (2019). Financing Global Common Goods for Health: When the World is a Country. *Health Systems & Reform*.

[91] World Health Organization (WHO) (2019). A Healthier Humanity: The WHO Investment Case for 2019-2023.

[92] Ornell F., Schuch J. B., Sordi A. O., Kessler F. H. P. (2020). “Pandemic Fear” and COVID-19: Mental Health Burden and Strategies. *Brazilian Journal of Psychiatry*.

[93] Carter A., Msemburi W., Sim S. Y. (2024). Modeling the Impact of Vaccination for the Immunization Agenda 2030: Deaths Averted Due to Vaccination Against 14 Pathogens in 194 Countries From 2021-2030. *Vaccine*.

[94] Marani M., Katul G. G., Pan W. K., Parolari A. J. (2021). Intensity and Frequency of Extreme Novel Epidemics. *Proceedings of the National Academy of Sciences*.

[95] World Health Organization (WHO) (2022). *A Healthy Return: Investment Case for a Sustainably Financed WHO*.

[96] Sheehan P., Rasmussen B., Sweeny K., Maharaj N., Symons J. (2022). *WHO Investment Case 2.0: Technical Report*.

[97] Cardoso T. A., Navarro M. A., Soares B. E., Silva F. L., Rocha S. S., Oda L. M. (2005). Memories of Biosafety in Brazil: Lessons to Be Learned. *Applied Biosafety*.

[98] Federal Government of Brazil Law No. 11.105 of March 24, 2005 (Biosafety Law) (Courtesy translation provided by WIPO). https://www.wipo.int/wipolex/en/legislation/details/8300.

[99] Campos A. S., Mclntosh D., Granjeiro J. M. (2018). *Biossegurança Laboratorial: Consolidação e Harmonização Das Normas Brasileiras*.

[100] Borre F., Borri J. I., Cohen Y. Z., Gasparoto M., Gurung T. B. (2022). Impact of the COVID-19 Pandemic on Infectious Diseases in Brazil: a Case Study on Dengue Infections. *Epidemiologia*.

[101] Mendonça A. D., Zuelke K. A., Kahl-Mcdonagh M. M., Mafra C. (2024). Comparison of Brazilian High- and Maximum-Containment Laboratories Biosafety and Biosecurity Regulations to Legal Frameworks in the United States and Other Countries: Gaps and Opportunities. *Applied Biosafety*.

[102] https://www.gov.br/mcti/pt-br/acompanhe-o-mcti/noticias/2023/08/brasil-tera-primeiro-laboratorio-de-maxima-contencao-biologica-do-mundo-conectado-a-uma-fonte-de-luz-sincrotron.

[103] de Mello Malta F., Amgarten D., Nastri A. C. S. S. (2020). Sabiá Virus–Like Mammarenavirus in Patient With Fatal Hemorrhagic Fever, Brazil, 2020. *Emerging Infectious Diseases*.

[104] National Center for Research in Energy and Materials (CNPEM) Brazil Will Have the World’s First Maximum Biosafety Containment Laboratory Complex Connected to a Synchrotron Light Source. https://cnpem.br/en/brasil-tera-nb4-conectado-sincrotron-mundo/.

[105] Brazil. Ministry of Health Ordinance No. 1.736, of November 8, 2023. https://bvsms.saude.gov.br/bvs/saudelegis/gm/2023/prt1736_09_11_2023.html.

[106] World Health Organization (WHO) (2005). International Health Regulations. https://iris.who.int/bitstream/handle/10665/246107/9789241580496-eng.pdf?sequence=1.

[107] Ellwanger J. H., de Lima Kaminski V., Chies J. A. (2019). Emerging Infectious Disease Prevention: Where Should We Invest Our Resources and Efforts?. *Journal of Infection and Public Health*.

